# Complete Genome Sequences of Two WU Polyomaviruses Detected in Pediatric Patients with Fatal Respiratory Infection

**DOI:** 10.1128/MRA.00052-21

**Published:** 2021-09-09

**Authors:** Hongwei Zhao, Qianyu Feng, Xiaohui Wang, Yingchao Liu, Suyun Qian, Lili Xu, Zhengde Xie

**Affiliations:** a Beijing Key Laboratory of Pediatric Respiratory Infection Diseases, Key Laboratory of Major Diseases in Children, Ministry of Education, National Clinical Research Center for Respiratory Diseases, National Key Discipline of Pediatrics (Capital Medical University), Beijing Pediatric Research Institute, Beijing Children’s Hospital, Capital Medical University, National Center for Children’s Health, Beijing, China; b Department of Pediatric Critical Care Medicine, Beijing Children’s Hospital, Capital Medical University, National Center for Children’s Health, Beijing, China; c Research Unit of Critical Infection in Children, Chinese Academy of Medical Sciences, Beijing, China; KU Leuven

## Abstract

Here, we report the complete genome sequences of two WU polyomavirus (WUPyV) strains, both obtained in 2020 from pediatric patients with fatal respiratory infection in Beijing, China. The double-stranded DNA (dsDNA) genome sequences of BCH2008-1 and BCH2020_1 are 5,229 bp and 5,228 bp long, respectively.

## ANNOUNCEMENT

WU polyomavirus (WUPyV) was first described in 2007 and isolated from the respiratory tract samples from children suffering from acute respiratory infection (1). It belongs to the *Polyomaviridae* family and is also known as human polyomavirus 4 (2). WUPyV has a highly conserved circular double-stranded DNA genome. WUPyV is commonly found in children with acute respiratory symptoms or immunocompromised patients (3–5) and is often found in coinfection with other pathogens (6). Seropositivity for WUPyV in the adult population is more than 90% (7, 8), indicating the wide distribution of the virus. Despite the ubiquitous nature of WUPyV strains, the pathogenicity of the virus remains unclear. Here, we report the complete genome sequences of two WUPyV strains obtained from two pediatric patients with fatal respiratory infection in Beijing, China. This study was performed in strict accordance with human subject protection guidance provided by the Research Ethics Committee of Beijing Children’s Hospital, Capital Medical University.

Nasopharyngeal swabs were collected from two children in March and April 2020 and subjected to metagenomic analyses. Nucleic acids were extracted from each sample using the Direct-zol RNA miniprep kit (Zymo Research, Irvine, CA, USA) and TRIzol LS reagent (Thermo Fisher Scientific, Carlsbad, CA, USA). The DNA/RNA concentrations were measured using a Qubit fluorometer (Thermo Fisher Scientific). A Nextera XT library (Illumina, San Diego, CA) was constructed, and the quality of the DNA libraries was assessed using a Qsep1 biofragment analyzer (BiOptic, La Canada Flintridge, CA) to measure the sizes of fragments before sequencing. The quantified DNA libraries were pooled and sequenced on a NextSeq 550Dx sequencing platform (Illumina). A single-end (75-bp read length) sequencing strategy was used. The primary sequencing output was demultiplexed using bcl2fastq v2.17.1.14; the reads were quality trimmed and subsequently removed if shorter than 20 bases using Trimmomatic v0.32. Reads that passed these filters were mapped against human references using Bowtie v2.2.4. Reads that aligned to either of the references were removed. The remaining reads were subjected to a BLASTn v2.2.30 search, and mapping was performed using CLC Genomics Workbench v21.0.3. All tools were run with default parameters unless otherwise specified. Two complete genome sequences of 5,229 and 5,228 bp were identified after mapping 35,550 (29,374,647 reads in total; 0.12%) and 56,897 (37,864,592 reads in total; 0.15%) sequencing reads, respectively, against the 5,229-bp-long reference genome for WUPyV (GenBank accession no. NC_009539). In addition to WUPyV, reads mapping to human bocavirus 1 and rubella virus were detected in the first sample, whereas reads mapping to Epstein-Barr virus, human cytomegalovirus, and human coronavirus NL63 were identified in the second sample. The detailed analysis of these viruses falls outside the scope of this announcement.

**FIG 1:**
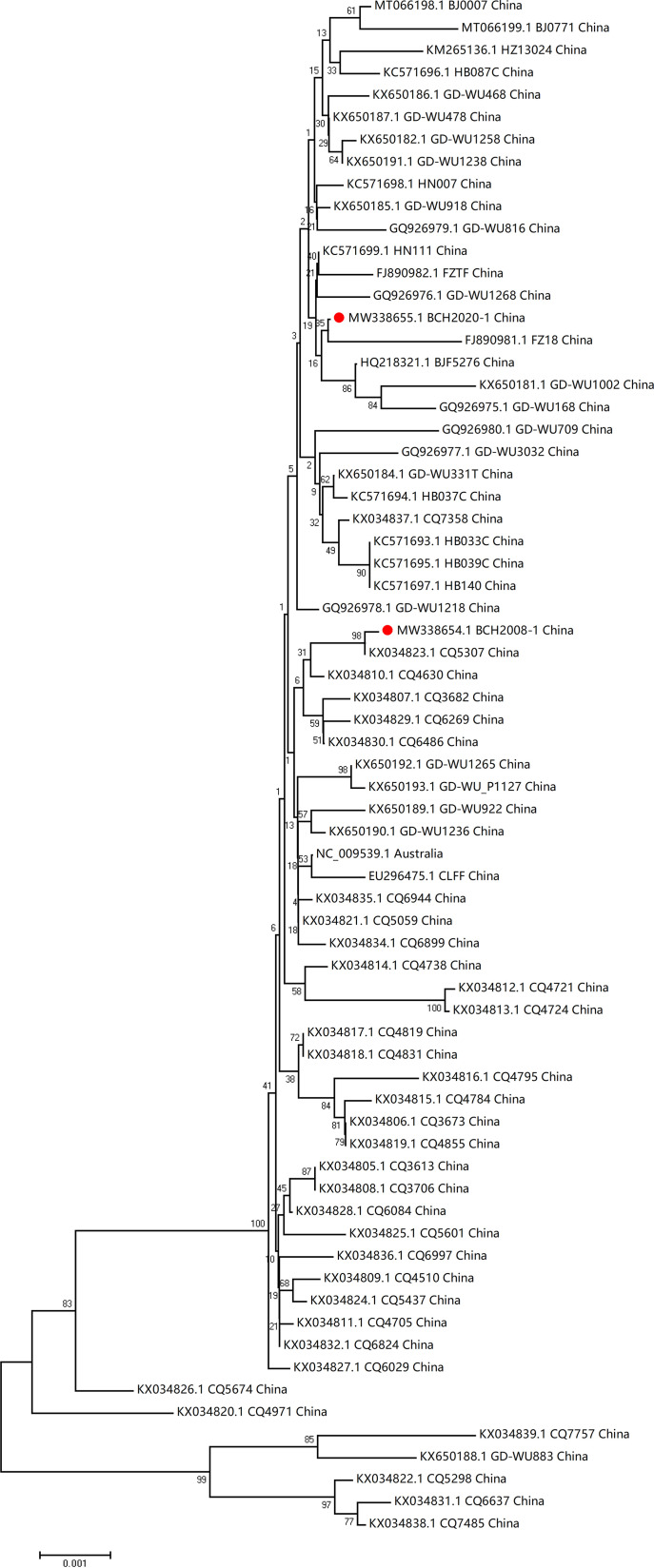
Phylogenetic tree based on the two WUPyV complete genome sequences. MEGA v7.0 software was used to generate phylogenetic trees with the neighbor-joining method and the Kimura 2-parameter model. The robustness of the phylogenetic trees was assessed using the bootstrap method with 1,000 replicates. Reference genomes with bootstrap values below 70 are not shown. The strains in the current study are marked with a solid red circle.

Compared with the genome submitted under GenBank accession no. NC_009539, there were 6 nucleotide mutations in BCH2008-1 and BCH2020_1, respectively, with 1 and 3 of them being nonsynonymous variations, (Table 1). Phylogenetic analysis revealed that BCH2008-1 and BCH2020_1 grouped most closely with strains CQ5307 (KX034823.1) and FZ18 (FJ890981.1), which were collected in 2013 and 2012, respectively, in China (Fig. 1).

**TABLE 1:** Nonsynonymous changes in the amino acids of two WUPyV strains, when aligned with the reference strain^*a*^

Strain	Accession no.	Nucleotide variant(s) for:		
		VP1	VP2	Large T antigen
BCH2008-1	MW338654		Glu250Gln	
BCH2020_1	MW338655	Ala82Thr	Met324Ile	Asn56Lys

a The reference strain can be found under GenBank accession no. NC_009539. Multiple nucleotide sequence alignments were performed using MAFFT software (https://www.ebi.ac.uk/Tools/msa/mafft/).

### Data availability.

The whole-genome sequences of two WUPyV strains have been deposited in GenBank under accession numbers MW338654 and MW338655. The raw sequence data from the metagenomic analyses of the two WUPyV strains are available at the NCBI SRA, under BioProject accession no. PRJNA741337.
